# Factors associated with adherence to post-treatment follow-up among a cohort of women with acetic acid/Lugol’s iodine positive lesions of the cervix in Cameroon: A retrospective cohort study

**DOI:** 10.1016/j.gore.2023.101269

**Published:** 2023-09-09

**Authors:** Simon M. Manga, Joseph F. Nkfusai, Kathleen L. Nulah, Florence Manjuh, Joel Fokom-Domgue, Yuanfan Ye

**Affiliations:** a Women’s Health Program, Cameroon Baptist Convention Health Services, Bamenda, P.O. Box 1, Nkwen, Bamenda, Cameroon; b Center for Women’s Reproductive Health, Department of Obstetrics & Gynecology, University of Alabama at Birmingham, 1700 6th Avenue South, Suite 10270, Birmingham, AL 35233, USA; c College of Nursing and Health Sciences, University of Massachusetts Boston, 100 Morrissey Boulevard, Boston, MA 02125-3393 USA; d Department of Public Health and Hygiene, Faculty of Health Sciences, University of Buea, P. O. Box 63, Buea, Cameroon; e Division of Cancer Prevention and Population Sciences, The University of Texas M.D Anderson Cancer Center, 1155 Presser Street, Houston, TX, 77030, USA

**Keywords:** Follow-up study, Acetic acid/Lugol’s solution, Cameroon, Uterine cervical neoplasms, Precancerous conditions, HIV seropositivity

## Abstract

**Background:**

Women treated for cervical precancerous lesions have up to a 30 % increased risk of developing cervical cancer compared to women in the general population. The aim of this study was to identify predictors for adherence to follow-up among women treated for precancerous lesions of the cervix in Cameroon.

**Materials and Methods:**

The study design was a retrospective cohort analysis of a five-year follow-up for women in Cameroon who were initially treated for cervical precancer lesions in 2013. Logistic regression models were used to determine factors associated with adherence to post-treatment follow-up. Statistical significance was set at p < 0.05.

**Results:**

Of the 344 women treated in 2013, 154 (44.77 %) never returned for a single post-treatment follow-up in five years. Marital status was the only variable statistically significantly associated with 5-year post-treatment follow-up adherence. women who had ever been married were 0.36 times (0.14 0.93)); p = 0. 0.035] less likely to adhere to post-treatment follow-up compared to women who have never been married. Although age was not statistically significant, women in the age group 30–49 years had some significance and they were 60 % [aOR, 95 %CI: 0.40 (0.18 0.89); p = 0.024] less likely to adhere to post-treatment follow-up when compared to women who were<30 years.

**Conclusion:**

Only about half of the women treated for cervical precancer in this cohort returned for post-treatment follow-up. Conducting needs assessments among these populations that are less likely to adhere to follow-up will allow us to implement and test strategies to improve adherence to follow-up.

## Highlights

• Adherence to post-treatment follow-up among women treated for cervical precancer is not optimal.
• Only about half of the women treated for cervical precancer in our cohort returned for post-treatment follow-up.
• Marital status was significantly associated with odds of adherence to post-treatment follow-up.

## 1 Background

Cervical cancer is one of the most preventable cancers. It begins with a precancerous lesion or a pre-invasive stage of the disease which, even though asymptomatic, can usually be diagnosed through screening (Wright et al., 2014). Treatment for cervical precancer is very effective with a cure rate of almost 100 % (Cox et al., 2014). However, treating cervical precancerous lesions does not completely eliminate the risk of developing invasive cervical cancer (ICC). Women treated for cervical precancers have up to a 30 % increased risk of developing ICC compared to women in the general population (Aerssens et al., 2008, Bae et al., 2007, Oga et al., 2016). This increased risk is even higher among women living with HIV where treatment failure is up to 51 % for ablation-eligible lesions and 55 % for excision-eligible lesions (Reimers et al., 2010).

Thus, post-treatment follow-up is recommended for women treated for Visual Inspection with Acetic Acid (VIA) and visual inspection with Lugol’s iodine (VILI) positive lesions (precancerous lesions of the cervix), and the interval and the number of follow up depend on the type of treatment (ablative therapy versus excision) and the completeness of the treatment. Typically, the World Health Organization (WHO) recommends that women treated for screen-positive lesions in a screen-and-treat approach, be followed up yearly until two consecutive negative screens are obtained (World Health Organization, 2014). Despite these recommendations, a significant proportion of women treated for screen-positive lesions do not show up for post-treatment follow-up (Ezechi et al., 2014). In order to achieve the WHO strategy to eliminate cervical cancer by 2030, adherence to post-treatment follow-up of women treated for precancerous lesions of the cervix (who are at higher risk for ICC) needs to be prioritized. The burden of non-adherence to post-treatment follow-up among women with cervical precancer can have an impact on cervical cancer morbidity and mortality in Sub-Saharan Africa (SSA) (Ezechi et al., 2014) where the burden of cervical cancer is high.

The importance of adherence to post-treatment follow-up among women treated for cervical precancer is to identify recurrent or persistent lesions to re-treat them in a timely manner to prevent progression to ICC. Therefore, programs that screen and treat women for precancerous lesions of the cervix without a mechanism in place to assure their post-treatment follow-up, might not be contributing optimally to the cervical cancer elimination call. Post-treatment lesion recurrence is even higher in women living with HIV compared to their HIV-negative counterparts (De Vuyst et al., 2014, Omenge Orang’o et al., 2017). Treatment using Large Loop Excision of the Transformation Zone (LLETZ) has lower post-treatment lesion recurrence/persistence compared to cryotherapy/thermal ablation (TA) (Oga et al., 2016, De Vuyst et al., 2014, Omenge Orang’o et al., 2017, Huchko et al., 2014, Del Mistro et al., 2015).

The Cameroon Baptist Convention Health Services (CBCHS) runs the most comprehensive cervical cancer prevention program in Cameroon and the Central African Sub Region called the Women’s Health Program (WHP) (DeGregorio et al., 2016). WHP follows WHO guidelines that recommend follow-up of all women treated for cervical precancer yearly until two consecutive negative screens are obtained. Post-treatment follow-up analysis in one of the WHP sites, Mboppi Baptist Hospital Douala, in Cameroon’s Economic capital city, revealed only 20 % of 290 women treated had post-treatment follow-up at one year, with a drastic decrease to 0.7 % at year two and three post-treatment *(Unpublished doctoral thesis. Akago SC. Lésions précancéreuses du col de l’utérus: Diagnostic prise en charge et evolution à l’Hôpital Baptiste de Mboppi [Precancerous Lesions of the Cervix: Diagnostic management and Follow-up in Mboppi Baptist Hospital]. Cameroon: University of Douala; 2017).* Manga and colleagues conducted a qualitative pilot study among the nurses and patients of WHP to explore the barriers to post-treatment follow-up. The main barriers that emerged were forgetting appointments, distance, cost, fear, level of education, marital status, and occupation Manga et al. (2019), and this formed the basis for our hypotheses in this study.

We previously published data on a cohort of screened women evaluating predictors of receiving recommended treatment for VIA/VILI-positive cervical lesions (either on the same day or at a follow-up visit) (Manga et al., 2020). Of the 755 women with cervical precancer lesions, only 344 (45.6 %) received treatment. This is a follow-up study that focuses upon the longer-term follow-up of the subset of women who were treated to determine the rate and predictors of adherence to post-treatment follow-up as this subgroup is at particular risk for lesion persistence, recurrence, or progression to ICC. The purpose of this study was to examine the rate of follow-up within five years and predictors associated with adherence to post-treatment follow-up among women treated for VIA/VILI positive lesions (cervical precancer) in Cameroon.

## 2 Materials and methods

The study design was a retrospective cohort analysis of 344 women who were treated for VIA/VILI-positive lesions in 2013 in Cameroon. Five-year post-treatment follow-up was conducted retrospectively for this cohort through 2018. Data were abstracted from electronic and paper medical records. The study received Institutional Review Board approval from the Cameroon Baptist Convention Health Services (CBCHS) and the University of Massachusetts Boston (UMB). The initial study population and procedure have been described in our previous work (Manga et al., 2020).

We developed a checklist and data codebook using the factors that pertain to follow-up using a systems model (Manga et al., 2020), and the WHP data manager used these resources to develop a dataset with the variables. In cases of missing data in the database, clinic registers were consulted. The dataset was then de-identified for statistical analyses. Results of the findings were represented in two tables: Participants’ Characteristics at Enrollment According to Post-Treatment Follow-Up and Logistic Regression of Predictors for Adherence to Post-Treatment Follow-up (N = 344). In these tables, a distinction was made between the percentage of post-treatment follow in LLETZ and in cryotherapy/TA.

### 2.1 Measures

All the predictors were those collected at enrollment. The independent variables were classified into personal, health status, and environmental variables. The personal variables were: age (<30, 30–49, and ≥ 50 years old); level of education (0 to 7yrs, 8 to 14yrs, and ≥ 15 years); marital status: [never married, and ever married (including the widowed, separated and divorced)]; employment status (unemployed versus employed); out of pocket payment for treatment (paid versus not paid); health status variables were the VIA/VILI lesion characteristics (eligible for ablation versus eligible for excision) and HIV status (negative versus positive). The environmental variable was coded based on distance (whether the woman lived out of the city where the clinic was located versus within the city); and type of clinic (mobile versus stationary).

The dependent variable for this study was adherence to post-treatment follow-up. Adherence was classified into two levels: adherent and non-adherent. Adherent patients referred to any woman treated for VIA/VILI positive lesions who observed at least one post-treatment follow-up appointment in any WHP clinic within five years post-treatment. Non-adherent patients referred to any woman treated for VIA/VILI lesion for whom no post-treatment follow-up appointment was reported in the WHP five years after the treatment.

### 2.2 Statistical analysis

The association between each independent variable (personal, health status, and environmental variables) and the dependent variable (post-treatment follow-up) was examined in a two-way table with measures of association (Pearson’s chi-square) and logistic regression in STATA version 17. In Pearson’s chi-square, an analysis of the association between each independent variable and the dependent variable was done to determine significance. In the logistic regression, a bivariable logistic regression was done for each independent variable and dependent variable to determine the crude odds ratio and significance. Variables with significant (p < 0.05) crude odds ratios were entered into multi-variable regression to determine the adjusted odds ratio with a 95 % confidence interval. The summarized outputs were presented in tables bearing odds ratios with p-value and 95 % CI.

## 3 Results

**Figure 1:**
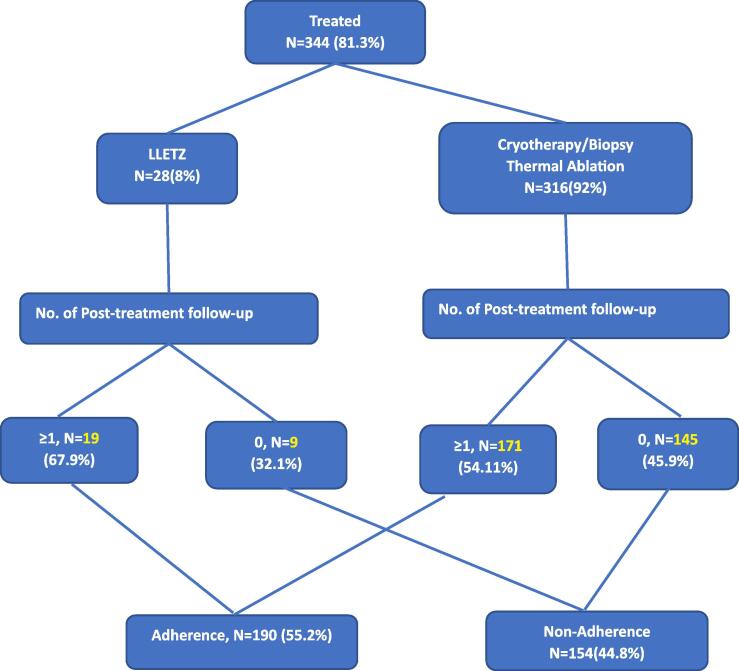
Cervical precancer treatment algorithm.

**Figure 2:**
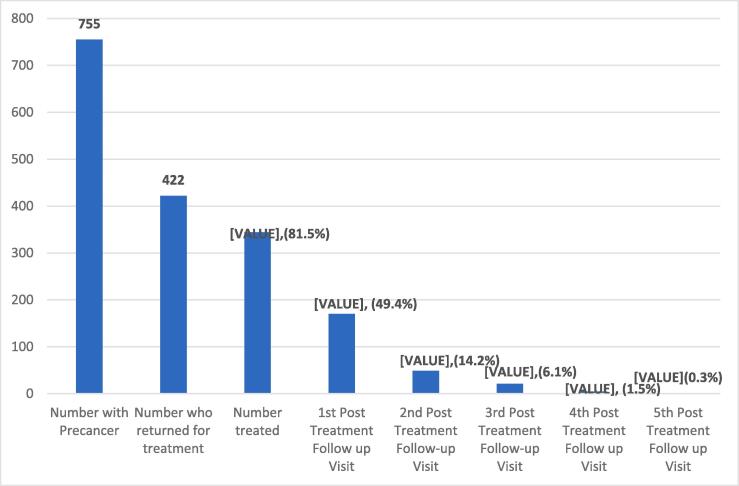
Five year follow-up trend among women with cervical precancer in Cameroon.

Of the 344 women treated for VIA/VILI lesions, 154 (44.8 %) never returned for a single post-treatment follow-up visit in five years and were classified as non-adherent (Figure 1). In the adherent group of 190 women, 114 women (60 %) returned for only one follow-up visit (11 (9.6 %) who had received LLETZ and 103 (90.4 %) cryotherapy/TA. The 76 women who attended two or more post-treatment follow-up visits included 10 (13.2 %) who had LLETZ and 66 (86.8 %) who had cryotherapy. Figure 2 presents a general trend from diagnosis to post-treatment follow up showing a drastic drop at every level of care from diagnosis to treatment to post-treatment follow-up. Table 1 shows the general characteristics of participants included in the cohort.

**Table 1:** Table 1. Participants’ Characteristics at Enrollment According to Post-Treatment Follow-Up (N=344)

Characteristics	Post Treatment Followed Up (N = 190)	No Post Treatment Followed Up (N = 154)	Total	p-value
	n (%)	n (%)		
**Age in Years (M = 35.76, Sd = 8.06, Min = 19, Max = 68)**				
<30	31 (16.32)	43 (27.92)	74	**0.015**
30–49	151 (79.47)	101 (65.58)	252	
>50	8 (4.21)	10 (6.49)	18	
Total	190 (100)	154 (100)	344	

**Education Level -Years (M = 9.18, Sd = 4.41, Min = 0, Max = 22)**				
1–7 (Primary)	101 (55.49)	76 (52.41)	177	0.618
8 = 14 (Secondary)	55 (30.22)	51 (35.17)	106	
> 15 (Tertiary)	26 (14.29)	18 (12.41)	44	
Total	182 (100)	145 (100)	327	

**Marital Status**				
Never Married	26 (13.76)	35 (22.73)	61	**0.031**
Ever Married	163 (86.24)	119 (77.27)	282	
Total	189 (100)	154 (100)	343	

**Employment**				
Unemployed	114 (60.64)	85 (56.29)	199	0.419
Employed	74 (39.36)	66 (43.71)	140	
Total	188 (100)	151 (100)	339	

**Out of pocket Payment for treatment**				
Paid	59 (66.29)	33 (50.00)	92	**0.041**
Not paid	30 (33.71)	33 (50.00)	63	
Total	89 (100)	66 (100)	155	

**Lesion-Grade Characteristics**				
Ablation eligible	152 (80)	116 (75.32)	268	0.299
Excision eligible	38 (20)	38 (24.68)	76	
Total	190 (100)	154 (100)	344	

**HIV Status**				
Negative	39 (20.53)	37 (24.03)	76	0.437
Positive	151 (79.47)	117 (75.97)	268	
Total	190 (100)	154 (100)	344	

**Site of Screening**				
Stationary Clinic	118 (62.11)	91 (59.09)	209	0.569
Mobile Clinic	72 (37.89)	63 (40.91)	135	
Total	190 (100)	154 (100)	344	

**Distance**				
Live Within the City	75 (39.89)	61 (39.87)	136	0.996
Live Out of the City	113 (60.11)	92 (60.13)	205	
Total	188 (100)	153 (100)	341	

### 3.1 Personal factors

The chi-square index however had a significant association (**p** = **0.041**) between fee payment and adherence to post-treatment follow-up. Other personal factor variables including educational level, and employment, were not statistically significant and were not included in the multivariate model.

Marital status was statistically significant. Although the age variable was not significant, the age group 30–49 had some significance. After controlling for level of education, employment status, HIV status, lesion grade, and site for screening, women in the age group 30–49 years were 60 % [aOR,95 %CI: 0.40 (0.18 0.89); p = 0.024] less likely to adhere to post-treatment follow up when compared to women who were <30 years and the women who had ever been married were 64 % [0.36 (0.14 0.93)); p = 0. 0.035] less likely to adhere to treatment compared to women who had never been married. Women who paid for their treatment completely were 1.9 times [aOR,95 %CI: 1.88 (0.95 3.72), p = 0.068] more likely to adhere to post-treatment follow-up when compared to those who did not pay or only paid part of the treatment fee even though this was not significant (Table 1, Table 2).

**Table 2:** Table 2. Logistic Regression of Predictors for Adherence to Post-Treatment Follow-up (N=344)

Predictors	cOR (95 %CI)	p Value	aOR (95 %CI)	P Value
**Age (Years)**				
<30	(Ref)			
30–49	0.48 (0.28 0.82)	**0.007**	0.40 (0.18 0.89)	**0.024**
>50	0.90 (0.32 2.54)	0.844	0.40 (0.06 2.60)	0.338

**Education Level (Years)**				
1–7 (Primary)	(Ref)			
8 = 14 (Secondary)	1.23 (0.76 2.0)	0.397	–	–
> 15 (Tertiary)	0.92 (0.47 1.80)	0.808	–	–

**Marital Status**				
Never Married	(Ref)			
Ever Married	0.54 (0.31 0.95)	**0.032**	0.36 (0.14 0.93)	**0.035**

**Employment**				
Unemployed	(Ref)		–	–
Employed	1.20 (0.77 1.85)	0.419	–	–

**Out of pocket Payments**				
Paid	(Ref)		–	–
Not paid	1.97 (1.02 3.78)	**0.042**	1.88 (0.95 3.72)	**0.068**

**Lesion-Grade Characteristics**				
Low	(Ref)		–	–
High	1.31 (0.79 2.18)	0.299	–	–

**HIV Status**				
Negative	(Ref)		–	–
Positive	1.22 (0.73 2.04)	0.437	–	–

**Site of Screening**				
Stationary Clinic	(Ref)		–	–
Mobile Clinic	1.13 (0.73 1.75)	0.569	–	–

**Distance**				
Live Within the City			–	–
Live Out of the City	1.00 (0.65 1.55)	0.996	–	–

### 3.2 Health Status Factors

All the health status factors; HIV status and lesion grade characteristics were not statistically significant and were not included in the multivariate model.

### 3.3 Environmental Factors

Distance and place of screening (stationary or mobile clinic) were also not statistically significant and were not included in the multivariate model (Table 2).

## 4 Discussion

Only half of the 344 women in our cohort made at least one post-treatment follow-up visit in five years. In a recent US study of adherence to post-treatment follow-up of women who received cryotherapy for precancer of the cervix, the adherence was 60.1 % (95 %CI: 51.6, 68.2) (Lendrum et al., 2022) slightly higher than what we found. Yet, the US study was run just for a year. Perhaps, the proportion would have been higher than 60 % had it been they followed up the women for a few more years. However, one would have expected a way higher than 60 % adherence in the US given their well-organized healthcare system. This is a pointer that adherence to post-treatment follow-up of women treated for precancer of the cervix might not only be a problem in low-and-middle-income countries (LMICs) but also in high-income countries (HICs). In this era when WHO is strategizing to eliminate cervical cancer worldwide, adherence to post-treatment follow-up for precancerous lesions of the cervix has to be given utmost importance. Therefore, strategies to improve adherence to post-treatment follow-up of cervical precancer have to be considered more broadly, and geographically.

Concerning personal factors, marital status was statistically significant, but age was not statistically significant except for the age group 30–49 which had some statistical significance to post-treatment follow-up in this study. In our previous work, older age was the most significant predictor of VIA/VILI-positive lesion treatment uptake. For example, we found there was treatment uptake of 83 % of women aged 30–39 compared to 61 % of women less than age 30 (aOR = 1.61, p = 0.006, 95 % CI 1.14–2.26) (Del Mistro et al., 2015). There have been conflicting findings between age and adherence to post-treatment follow-up in some studies. For example, in a cross-sectional study in the U.S., women over 50 years were more likely to be adherent to post-treatment follow-up than women younger than 50 (RR: 1.08, 95 %CI 0.88, 1.32) (Lendrum et al., 2022). Meanwhile, in a cross-sectional study in Botswana, older age was not associated with adherence to follow-up (Coker et al., 2007). However, the Botswana study was looking at re-screening adherence including both women who were negative and positive for cervical precancer who were living with HIV. Other studies have found associations between personal factors; older age, higher educational level, being employed, and being married with adherence to post-treatment follow-up (Barchi et al., 2019, Fish et al., 2013, Hui et al., 2014). In our prior qualitative pilot study, which was the basis for our hypotheses in this study, we found that women who pay for their treatment upfront are most likely to be adherent to post-treatment follow-up than women who do not pay for their treatment upfront potentially due to the guilt of owing the hospital. (Manga et al., 2019) Even though this study indicates that those who did not pay for their services were less likely to adhere to post-treatment follow-up, the association was not statistically significant in the logistic regression model. Another study found associations between adherence to post-treatment follow-up and cost (Khalid et al., 2011). However, payment of services is not captured in the WHP database, so the information was extracted manually from paper records, which may not have been accurate. It is therefore important to include payment information in the database, so that a future study should examine the impact of treatment payment on follow-up adherence.

Regarding health status factors, contrary to our hypothesis that women living with HIV will be more adherent to post-treatment follow-up than their HIV-negative counterparts, we did not find any statistical significant association between HIV status and adherence to post-treatment follow-up. HIV-positive women usually have frequent hospital appointments, and one would expect that their general adherence to all medical appointments would be higher compared to their HIV-negative counterparts. However, the HIV status in our study was self-reported, which is a limitation of the study. The other health status factor, lesion grade characteristics, was not statistically significantly associated with adherence to post-treatment follow-up in this study. In line with this, a U.S. study also did not find any association between adherence to post-treatment follow-up and higher-grade lesions (Maza et al., 2016).

Concerning environmental factors, there was no association between mobile and stationary clinics and post-treatment adherence. Usually, mobile clinics are conducted in very remote areas with poor road networks and transport systems creating a barrier for these women to return for follow-up (Manga et al., 2019). A strategy needs to be developed to address the follow-up of women screened in mobile clinics. The best strategy could be to have the staff revisit each community where a mobile clinic was done after a year to follow up on those women who were treated. Thus far, this has not been practical because WHP is a self-sustained program. Traveling several miles to follow up a handful of women has not been justified in regard to cost and human resources. Some strategy has to be established to encourage these women to return for their post-treatment follow-up visits at the stationary clinics. One possible solution is to utilize mobile reminders but unfortunately, most remote settlements in Cameroon do not have telephone network services. Thus, the most practical solution is to provide follow-up screening at half the normal cost.

There was no significant association in this study between living within the city or out of the city and adherence to post-treatment follow-up. This contradicts a Nigerian study where women who lived more than 10 km from the clinic were 260 % more likely to follow up for treatment than those living further (OR: 3.6, 95 %CI:1.5–9.1) (Oga et al., 2016). However, this study was focused on treatment follow-up and not post-treatment follow-up. Notwithstanding, in our previous study on treatment follow-up, we still did not find any association between distance and treatment uptake (Manga et al., 2020).

The major strength of this study is that it is one of the first studies to evaluate post-treatment follow-up of a cohort of women treated for cervical precancer lesions in a resource-limited setting.

### 4.1 Limitations

This study had a number of limitations: 1) It is a review of medical records with data that was primarily collected for clinical work and program evaluation, and not for the purpose of research 2) The HIV status was self-reported. For reasons that might be associated with stigma, some HIV-positive patients might have reported their status as negative. Thus, creating a potential bias towards the null 3) The dataset did not have some aspects of the systems model that could affect post-treatment follow-up up 4) The reasons for non-adherence to follow-up were not assessed. It is possible that some patients did not follow up because they died or traveled abroad but this information was not available 5) Though our program is the largest and most comprehensive cervical cancer prevention program in Cameroon, it was still possible that some women went for their posttreatment follow up to other cervical cancer prevention programs.

### 4.2 Conclusion

Marital status was significantly associated with odds of adherence to post-treatment follow-up. Conducting needs assessments among these populations that we found are less likely to adhere to follow-up will allow us to implement and test strategies to improve adherence to follow-up. Cervical cancer screening clinics need to keep a register of women who have been treated to ensure that they are not lost to follow-up. The clinics should collaborate with HIV care and treatment clinics to ensure that women living with HIV who are treated for cervical precancer receive appropriate follow-up since they are more at risk of treatment failure.

To better understand this phenomenon, longitudinal prospective studies are needed to examine predictors for adherence to post-treatment follow-up among women with cervical precancer lesions in Cameroon and SSA. Future studies should also consider investigating the reasons for poor post-treatment follow-up.

## Funding

This research received no external funding.

**Informed Consent:**

The IRB waived requirements for informed consent because the study was based on review of secondary data.

**Institutional Review Board (IRB) Statement:**

This research was granted expedited and except approval by the Cameroon Baptist Convention Health Services #IRB2021-04 and an IRB waiver by the University of Massachusetts Boston # 2018256.

**Informed Consent Statement:**

Not applicable.

## CRediT authorship contribution statement

**Simon M. Manga:** Conceptualization, Methodology, Writing – original draft. **Joseph F. Nkfusai:** Formal analysis, Writing – review & editing. **Kathleen L. Nulah:** Data curation. **Florence Manjuh:** Writing – review & editing. **Joel Fokom-Domgue:** Methodology, Writing – review & editing. **Yuanfan Ye:** Formal analysis.

## Declaration of Competing Interest

The authors declare that they have no known competing financial interests or personal relationships that could have appeared to influence the work reported in this paper.
